# Clouding the Judgment of Domestic Violence Law: Victim Blaming by Institutional Stakeholders in Cambodia

**DOI:** 10.1177/0886260515588919

**Published:** 2015-06-15

**Authors:** Katherine Brickell

**Affiliations:** 1 University of London, Egham, UK

**Keywords:** victim blaming, domestic violence, law, stakeholder, Cambodia

## Abstract

This article examines victims’ purported complicity in the judicial failures of domestic violence law to protect them in Cambodia. It is based on 3 years (2012-2014) of research in Siem Reap and Pursat Provinces on the everyday politics of the 2005 “Law on the Prevention of Domestic Violence and the Protection of the Victims” (DV Law). The project questioned why investments in DV Law are faltering and took a multi-stakeholder approach to do so. In addition to 40 interviews with female domestic violence victims, the research included 50 interviews with legal and health professionals, NGO workers, low- and high-ranking police officers, religious figures, and local government authority leaders who each have an occupational investment in the implementation and enforcement of DV Law. Forming the backbone of the article, the findings from this latter sample reveal how women are construed not only as barriers “clouding the judgment of law” but also as actors denying the agency of institutional stakeholders (and law itself) to bring perpetrators to account. The findings suggest that DV Law has the potential to entrench, rather than diminish, an environment of victim blaming. In turn, the article signals the importance of research on, and better professional support of, intermediaries who (discursively) administrate the relationship between DV Law and the victims/citizens it seeks to protect.

## Introduction

The problem we have is not the law, but rather the victims themselves. (Anchali, female, 59-year-old deputy district leader)

An established body of research has highlighted the blame commonly placed by institutional stakeholders, perpetrators, and the media on women for the domestic violence (DV) they experience (Aisyah & Parker, 2014; Berns, 2001; Burgess, 2012; Koepke, Eyssel, & Bohner, 2014; Mitra, 2013; Thapar-Björkert & Morgan, 2010). This article extends such literature by qualitatively exploring women’s (supposed) complicity in the judicial failures of domestic violence law (DV Law) to protect them. Drawing on 3 years (2012-2014) of research in Cambodia on the 2005 “Law on the Prevention of Domestic Violence and the Protection of the Victims,” it demonstrates how law has the potential to extend, rather than alleviate, an environment of victim blaming. The rhetorics of institutional stakeholders central to the implementation and enforcement of DV Law form the backbone of this argument.

Providing better knowledge on, and understanding of, the rhetorical imposition of blame for inefficacies of DV Law is a timely priority. Despite feminist concerns voiced in the late 1980s about the “malevolence” of law and its embedded “masculine culture” (Smart, 1989, p. 2), the past 20 years have witnessed an unprecedented growth in the number and scope of laws that extend states’ duty to address violence against women (VAW), particularly in the developing world (United Nations Development Fund for Women [UNIFEM], 2009, 2010). Given this heavy emphasis on legal reform, United Nations Women (2011) reported that by 2011, 125 countries had outlawed DV specifically. Yet the organization also spotlights an apparent paradox that has arisen:

The past century has seen a “transformation” in women’s legal rights, with countries in every region expanding the scope of women’s legal entitlements. Nevertheless for most of the world’s women, the laws that exist on paper do not translate to equality and justice. (United Nations Women, 2011, np)

There is also mounting scholarly evidence that violations of women’s human rights remain unrelenting even in countries where legislative changes and political campaigns have been introduced to address VAW (see Frías, 2013, on Mexico; Hume, 2009, on El Salvador; Turbine, 2010, on Russia; Vetten, 2005, and Usdin, Christofides, Malepe, & Maker, 2000, on South Africa). The preface to the edited collection *Feminist Activism, Women’s Rights, and Legal Reform* speaks in a similar vein to the complexity of the legal arena as a space of domination and social transformation for women (Cornwall, 2013). Such ambivalence, verging on pessimism in some quarters, is balanced against continuing assertions about the potential of law in advancing women’s concerns (Lewis, Dobash, Dobash, & Cavanagh, 2001).

This article provides a locally articulated reading of victim blaming through the institutional stakeholder rhetorics that cast law-reneging victims as obstacles to DV alleviation in Cambodia. To date, existing research has tended to emphasize the influence of institutional actors on women’s reluctance to report men’s violence. As Dobash and Dobash (1992) report, such reticence is “often exacerbated by social, medical and legal institutions whose actions reveal a powerful legacy of policies and practices that explicitly or implicitly accept or ignore male violence” (p. 4). With recent exceptions (Briones-Vozmediano, Goicolea, Ortiz-Barreda, Gil-González, & Vives-Cases, 2014; Dunn & Powell-Williams, 2007; Lila et al., 2013), rather less research has concerned itself with the broader ramifications of institutional stakeholder perspectives and practices in cases where help *is* sought through DV Law. What Macaulay (2002) refers to as the “implementation gap” between “the letter and the application of the law” (p. 83) is directly mediated and controlled via these gatekeepers. They are service providers “who may observe, adapt, or completely ignore” DV Law and, in doing so, help or fundamentally hinder women’s access to justice (Briones-Vozmediano et al., 2014, p. 1008). Their professional behavior and conduct also send out powerful messages to victims, offenders, and wider society about the prospects of women gaining justice through the legal system (Lila et al., 2013). Dunn and Powell-Williams (2007) highlight in their U.S.-based research, for example, that (even) highly trained DV advocates “struggle to simultaneously conceive of battered women as victims trapped by social, psychological, and interactional forces as agents whose choices must be respected” (p. 977). Despite their empathetic approach, “the language available to them ultimately constructs the former in less-than-sympathetic terms” (Dunn & Powell-Williams, 2007, p. 978).

In Cambodia, it is recognized that “negative attitudes by judicial officers and law enforcement personnel towards women victims of violence continue to impede the effective prosecution of cases” (The Committee on the Elimination of Discrimination Against Women [CEDAW], 2013, p. 4). This article fleshes out this formalized and generalized statement. To do so, it positions law as a discursive mechanism by which “systems of meanings (and subsequent practices) about norms, rights, and identities” (Cornwall, 2013, p. 19) are internalized and operationalized by DV Law implementers and enforcers. The article elucidates the rhetorical devices by which police and local government officials divest responsibility for the abandonment of legal cases onto female victims. In these instances, DV Law is likely to entrench what Thapar-Björkert and Morgan (2010) identify as a dangerous ethos of blame and responsibility that contributes “to a culture in which violence is normalized, sustained, and ultimately accepted by default” (p. 47). In other words, although legal reform provisions a “new legal self protected from violence by men,” in practice, it is often a “protection never fully guaranteed or experienced” (Engle Merry, 2006, p. 188). As this article shows with respect to Cambodia, the promise of this “new legal self” actually has the potential to further castigate women who do not fully embrace it.

In the next section, the methodological background of the study is outlined before the article provides the Cambodian gender context. The empirics with the in-depth narratives of five male and female institutional stakeholders then follows. This limited number has been selected to reflect a range of perspectives identified as well as to provide a detailed account of participants’ backgrounds and related viewpoints. The discourses reveal the perceived emotional and submissive tendencies of Cambodian women, which retard the use of DV Law and render women blameworthy.

## Method

The insights provided in this article are based on 50 institutional stakeholder interviews transcribed and translated from Khmer into English verbatim (from digital voice recordings). They include interviews with legal and health professionals, NGO workers, low- and high-ranking police officers, religious figures, and local government authority leaders who each have an occupational investment in the implementation and enforcement of DV Law. Of these, 40 interviews were conducted equally between two case study provinces—Siem Reap and Pursat.^1^ Following WHO guidelines on the ethical conduct of research on DV, the provincial interview team included one male and one female research assistant who carried out the research after a period of in-province training, and piloting ran in collaboration with a gender-oriented NGO. The interviews with 24 men and 16 women represent the majority focus of the empirical analysis. Although the male interviewees worked predominantly as police officers, court officials, and community leaders, the female interviewees were mainly (albeit not exclusively) local counselors, deputy community leaders, and health professionals. A final 10 interviews in the country’s capital Phnom Penh were held with interviewees who had national-level oversight and responsibility. Together, they formed part of a larger suite of research joint on why investments in DV legal reform are faltering in Cambodia. In addition to a quantitative household survey element conducted with 1,177 lay participants over the age of 18, 40 interviews with female DV victims and a further 40 with male and female householders were conducted in both provinces (see Brickell et al., 2014 for full methodological information on the project). Participatory video workshops were also held in 4 of the communities (see Garrett & Brickell, 2015 for information on this specific element).

## Cambodian Gender Context

Since the turn of the millennium, the lack of success in converting legal reform advances into DV prevention has been acute in Cambodia. Nationally representative data show that in 12 months preceding a ground breaking Partners for Prevention (2013) study, 1 in 4 women reported at least 1 act of physical or sexual violence perpetrated by an intimate partner. The insidious nature of DV in the Southeast Asian nation has been a long-standing concern since inaugural research conducted in the mid-1990s (E. Nelson & Zimmerman, 1996; Zimmerman, 1995).^2^ A decade on, the 2005 progress report on plans to achieve Cambodia’s Millennium Development Goals (MDGs) benchmarked domestic violence as *the* major challenge to reaching targets of promoting gender equality (Ministry of Planning, 2005). Yet the government response has been slow and inadequate (League for the Promotion and Defense of Human Rights [LICADHO], 2006). Although Cambodia ratified the Convention on the Elimination of all forms of Discrimination Against Women (CEDAW) in 1992 and the Optional Protocol in 2001, it was not until September 2005 that its first ever DV Law was passed. Still, now, weaknesses of implementation and enforcement of this civil law mean that Cambodian women rarely have the ability to defend themselves and their interests (Amnesty International, 2010). The study thus speaks to the overarching criticism made by CEDAW (2013) of the “limited progress made in the prevention and elimination of violence against women” (p. 7) in the country.

Indeed, although the focus of this article is on discursive strategies of victim blaming by institutional stakeholders, the Economic and Social Research Council/Department for International Development (ESRC/DFID)-funded research uncovered an accompanying range of barriers. These included, but were not limited to, structural gender inequities such as women’s economic dependence on men, a weak rule of law environment, and inadequacies of financial and human resources to support DV Law training, implementation, and enforcement (see Author et al., 2014). They add weight to concern expressed, again by CEDAW (2013), at the absence of a comprehensive legal aid system provided by the state/Bar Association on top of a corrupt and unofficial fees system that negatively affects not only women’s access to justice but also public trust (see Burns & Daly, 2014, for similar concerns with respect to rape).

These obstacles combined with a moral preference for upholding the “harmony” of the family contribute to a distinct reluctance on the part of institutional stakeholders to practice DV Law through judicial means (Brickell, 2015). As the Ministry of Women’s Affairs (MOWA; 2014) explain, DV Law offers two routes for remedying DV—“alternative dispute resolution” (ADR) and the “judicial solution” (p. 66). The *modus operandi* appears to be the former—*samroh samruol*—a Cambodian term for local reconciliation that has the meaning to smooth over and seek harmony. This is ordinarily sought through a meeting orchestrated by a village or commune leader who tries to encourage compromise between parties to reach an agreement marked verbally or by a promissory note (*liket sanya*).^3^ DV Law condones this approach in cases that fall under the rubric of “mental/psychological or economic affected violent acts and minor misdemeanours” (Article 26; Royal Government of Cambodia, 2005). As the official glossary to the dictate reads, the practice is meant to facilitate the “communication process between quarrelling parties that aims at maintaining family life” (Royal Government of Cambodia, 2007, p. 11). The imperative placed on harmony, consensus, and the collective is so pervasive, however, that many victims who have experienced “criminal offences that are characterized as felonies or severe misdemeanours” (Article 17), such as bodily injury, are still pressured into reconciliation with the same husband, often on a repeated basis (Brickell, 2015). In such cases, authorities in charge cannot intervene to reconcile or mediate; rather, equivalent articles in the country’s Penal Code should be used to pursue a criminal conviction (Article 17). Reflecting the political nature of women’s rights claiming (Burgess, 2012), CEDAW (2013) has made overt reference to the state’s propensity to “*dispose* of cases of violence against women through mediation” (emphasis added). Although in the ESRC/DFID research, institutional actors were not unaware of the myriad and underlying constraints placed on the “judicial solution,” it was largely women who were held accountable for its under-use in comparison with ADR. Yet the notion that women are complicit in the abandonment of legal intervention is complicated by the fact that institutional stakeholders rarely present this route as within the realms of possibility to victims. This matters, as in DV Law, the “nearest authorities in charge have the obligation to urgently intervene” (Article 9).

Although the article works to unpack these little-studied dynamics further, existing research in Cambodia has established multiple grounds on which women are judged and the perpetration of DV initially justified. In spousal relationships, these revolved around gender norm violations including preparing unappetizing food, failing in motherly duties and housekeeping, being sexually unavailable, and arguing too much (Luco, 2002; Surtees, 2003; United Nations Development Program [UNDP] Cambodia and VBNK, 2010). Data from two surveys supplement such findings with insights drawn from 300 police (lower and higher ranking) and local authorities (district and communal; MOWA, 2005, 2009). Although there has been a discernable drop in the percentage of stakeholders between the 2005 baseline and 2009 follow-up survey who consider violence acceptable, it is notable that in the later survey, violence by husbands is (still) often justified when a wife challenges her husband’s dominance. When a wife argues with her husband, does not obey, or does not show him respect, it was found that respondents deemed knocking her on the head (77% in 2005/46% in 2009), throwing an object at her (75%/42%), or cursing (73%/57%) acceptable behavior (MOWA, 2005, 2009). Their apportionment of blame thereby has roots in women’s comportment and behavior in their marital lives.

Women’s past lives are also significant, however. Although Buddhism represents an ethos of nonviolence, “it promulgates the rather merciless law of karma, according to which your present life situation is the cumulative result of deeds in your previous incarnations” (Ovesen, Trankell, & Ojendal, 1996, p. 77; see also Bhuyan, Mell, Senturia, Sullivan, & Shiu-Thornton, 2005, on the significance of Karma for immigrant Cambodian DV sufferers). It is within this fatalistic framework and within the confines of prevailing gender norms that women are often encouraged to endure the violence they encounter. In the Cambodian context then, Kent (2011) writes that

the international rhetoric about empowering women and raising their awareness of their rights therefore needs to be scrutinized in light of the fact that real economic pressures in interplay with cultural factors may increase of the vulnerability of many women. (p. 406)

The idea that women should accept domestic violence is related, in part, to 19th century normative Cambodian poems such as the *Chbab Srei* (Rules for Women; see Brickell, 2011, for full exploration of these texts). The women’s code deals predominantly with obeying and respecting spouses by keeping “fire in the house.” This Cambodian Buddhist expression embodies the idea that to maintain a harmonious household, women are responsible for suppressing three fires of potential conflict connected with their relationships to parents, husbands, and “others.” Women should not bring fire from outside into the house, not take fire inside the house outside, and should take care not to spread or overheat fires. Given its casting of DV as a private family affair, the *Chbab Srei* is regularly highlighted as a barrier to the alleviation of this human rights abuse and reflects the interplay of cultural factors that Kent (2011) raises.

## Victims as Self-Imposed Barriers to Legal Redress

The empirical sections that follow focus on institutional stakeholder rhetorics that paint women as complicit in their ongoing victimhood by withdrawing from legal recourse. In these narratives, neither empathy for the suffering of the victim, the actions of perpetrators, nor the violation of human rights is declared. Rather, the ESRC/DFID findings highlight two interlinked discourses, which institutional stakeholders harness: first, the emotionality of women, which is deemed incompatible with the rationality of law, and second, women’s submissive tendencies, which render them answerable for a climate of impunity.

Both are dimensions of victim blaming that raise complex questions surrounding the agency of victims to take up legal help. Seminal work by Dunn (2005, 2010) ventures that women are judged accountable for their actions given cultural codes, which posit that all individuals have free will. As she elaborates,

When battered women are depicted as staying in or returning to their violent relationships, this violates the normative expectation that people ordinarily act in their own best interest, which rests on the assumption that they are free to do so. (Dunn, 2005, p. 4)

Yet victims’ oscillating use/rejection of DV Law manifests in practice from women’s disempowerment, a response to restricted legal options rather than expression of agency. As Stubbs (2002) acknowledges, “A more complex conception of agency, together with an understanding of the control based nature of DV, should work against easy assumptions that women’s agency is unconstrained” (p. 45).

Although institutional actors are not naïve to the restrictions imposed on victims’ “choices,” an overriding sense remains that it is individual women who are taking DV Law hostage. Women are constructing their *own* entrapment rather than the structural inadequacies and coercive social norms that condition their everyday lives. In Cambodia, access to justice presents a compelling set of risks to victims, from economic ruin to social stigmatization. Taking a capabilities approach makes clear that “securing a right to someone requires making the person really capable of choosing that function” (Nussbaum, 2005, p. 175). It requires viewing rights as economic and material entities (Nussbaum, 2005). In the context of Spain, for example, Briones-Vozmediano et al. (2014) write of the “anticipated failure” expressed by professionals whose work on legal cases with immigrant women is repeatedly undermined by victims’ economic dependence, limited knowledge of available resources, and loss of social support after leaving their country of origin.

Under such circumstances, it is important not to assume that the practices of institutional stakeholders are a product of free will or unbridled capabilities either. In Cambodia, local authorities operate within an environment of chronic under-resourcing, lack of legal knowledge, and poor training, which severely hinders their ability to assist victims. Although DV Law has been ratified at least, the article’s findings strongly indicate that the Cambodian government is not enabling women to receive meaningful help from local stakeholders. This is problematic given that the capabilities approach “insists that all fundamental entitlements require and deserve state action for their protection, and that all must be supported, or else basic justice, minimal justice, has not been done” (Nussbaum, 2005, p. 175). From a charitable standpoint, local stakeholders who form the article’s foundation have very little agency to change these conditions and, in turn, look to victims not only to prevent spousal violence in the first place but also to play an active and agentic role in pursuing the “judicial solution.”

### Emotional Dimensions of Victim Blaming

I think that there is an element of shame involved when you talk about emotion. Women, especially Khmer women, are taught to be very patient at all times. They are to take the abuse with gritted teeth and smile to the world simultaneously.

As a 43-year old female Committee for Women and Children member explains, despite the challenging familial circumstances which women often contend with, in Cambodia, it is orthodox for “Khmer women” to be socialized to suppress their emotions. In these instances, women may feel obligated, albeit with “gritted teeth,” to outwardly develop a veneer of happiness so as to keep “fire in the house.” Henceforth, rights claiming through the assertion of extra-domestic agency could be considered transgressive of this gender reference.

The rebuttal of judicial assistance was commonly discussed by institutional stakeholders through reference to women who “bail out” their husbands on arrest or imprisonment. Kamol, a 49-year-old male police officer, is one such example. His interview also spoke to the difficulties faced in bringing cases to court. During his 6 years of dealing with DV cases, all but two stopped at local reconciliation (this example mirrors a general trend that formally adjudicated cases of DV are rare). Kamol’s analysis was typical of many institutional stakeholders: an initial recognition paid to the constraints women encounter, followed by a more piecing critique of victims’ complicity in the failure of their cases to progress beyond community confines.

Women face financial difficulties without their husbands around. Most of these women depend entirely on their spouses for financial support. Therefore, they have no choice sometimes but to seek the release of these men so that they can provide the family with financial support. Another important factor is the pressure these women face inside the community. The elders, along with their family, tend to force them to forgive the wrongdoings their spouses commit, claiming that it is natural that men are violent and mistake-prone.

But truthfully . . . it is always women themselves who cloud the judgment of the law. The fact that these women consistently bail out their abusive spouses has everything to do with the unhealthy emotional attachments they have with these violent men. Initially, when they are angry because of the abuse, they come over and are adamant about getting their spouses arrested. Later, as we try to do our jobs to bring them justice, they change their minds. They beg and cry for these men to be released . . . . the law is here; the question is whether or not the victim will embrace it. The law does its work by arresting her abusive husband. The legal procedure demands that he stands trial and faces punishment for his actions. The victim breaks this line of justice by demanding the court to release him and strip him of all personal responsibility . . . we are trying to be as lenient as legally allowed. However, the issue is not with us but rather with the victims themselves.

As evidenced in the first excerpt from Kamol’s interview, the limitations that compromise women’s full use of DV Law are identified on a range of fronts: economic dependence, the pressures of household survival, and the normalcy that kin and elders attach to DV. With the exception of motherhood, his interview shows an appreciation of the embedded nature of women’s intimate lives in familial and community relations, and their implications for women’s lack of options. Having said this, Kamol’s initial thoughts do not preclude the police officer from divesting majority blame onto victims. As evidenced in the second excerpt, Kamol goes on to provide a sustained critique of women’s excessive emotionality that contributes to the legal retraction of DV cases. The implication here is that women are too emotional for DV law to be effectively applied. As Leung (2014) writes in connection to her research with the police on DV in Hong Kong, “in the history of patriarchy, men’s speech has been regarded as rational, objective and right, whereas women’s speech has traditionally been ignored or considered worthless, especially by the criminal justice system” (p. 85). Associating femininity with irrationality, women’s indecision was also branded as one of the main reasons why officials concede on pragmatic grounds to (more “lenient”) local forms of reconciliation. Under these circumstances, victims’ behaviors bear witness perhaps less to their “unhealthy emotional attachments” as Kamol describes them, and rather more to the lack of alternatives that DV Law provides to remaining within abusive relationships. Moreover, women’s displays of “corporeal altruism” (Brickell & Chant, 2012, p. 153)—the sacrificing of bodily integrity and intimate security—could be borne out of a “trade off” between different dimensions of poverty and well-being (see Kabeer, 1997). The eschewing of legal help and return to a violent marriage may represent one such tactical compromise on account of factors beyond victims’ immediate control.

“Truthfully,” according to Kamol, however, “it is always women themselves who cloud the judgment of the law” by distorting proceedings. This perspective ignores the risks to women of accessing justice (which he himself is conscious of); the cycles of manipulative contrition and violence, which characterize DV; and the fact that love and marriage are, by their very nature, emotionally charged affairs. Perpetrator impunity nevertheless becomes conceived of as the victim’s fault in the police officer’s interview: “The victim breaks this line of justice by demanding the court to release him and strip him of all personal responsibility.” Despite the widely acknowledged impunity and opaqueness of the Cambodian law enforcement system (Walsh, 2007), law is measured akin to a line. Women by contrast blur the justice system and deny the agency of law to punish. Further interviews in the sample of interviewees demonstrate the rhetorical reliance on the emotionality of women to explain the environment of impunity surrounding DV Law. As a 69-year-old female district leader comments,

The biggest problem is the victims themselves. It is just too hard to uphold the law when they consistently create loopholes for these perpetrators to get away from the law. Often the blame lies with the victims. They are the ones that hinder the law from prosecuting those culprits. They bribe and beg the local authority to release those guys from arrest. And to just separate them all the time is not viable, either . . . there should be a solution on the issue of the victim demanding the release of the perpetrator . . . there should really be a provision in the law about this particular issue.

Ponnleu is explicit that victims “consistently create loopholes for these perpetrators to get away from the law.” Despite the plethora of barriers affecting women’s ability to “follow through” on legal redress, she chooses to focus on victims’ manipulation of local officials via bribery tactics to secure spousal release. With the constraints on women’s ability to pursue DV Law distinctly missing in her analysis, the solution inferred is to make DV a “no drop” charge so that officials do not need to “separate them all the time.”

In Ponnleu’s belief, the irrationality of women warrants a policy that denies DV victims the option of having the discretion to withdraw a complaint once formal charges have been filed. In contrast to mandatory arrest in other country contexts, in Cambodia, a converse lack of intervention (beyond local reconciliation) prevails through systematic failures to arrest offenders, for charges to be filed, and for cases to be successfully prosecuted in the courts. The outcome, however, is not dissimilar with the numerous and differentiated needs of victims being ignored. Although the legislative enactment that Ponnleu proposes may remove many structural impediments to prosecuting perpetrators, operational practices and discursive manifestations of blame would likely remain unchanged (Corsilles, 1994). In fact, current DV Law already makes provision for repeated incidences of “severe” DV to be treated in this way. As Article 36 reads, “In case domestic violence has been repeated in violation of the penal law, the court shall charge the perpetrators in accordance with the penal procedures, even if there is a request from the victims again.” In reality, however, given the low salaries of public officials, poor training, and patron-style leadership, bribes are systematically used to leverage the dropping of criminal cases across Cambodia (Broadhurst & Bouhours, 2009; Feinberg, 2009).

### Submissive Dimensions of Victim Blaming

Khmer women are too submissive . . . they are too blindly loyal to their spouses. (Devi, male, 35-year-old provincial administrator)

Despite deep-seated persistent male biases that dictate that women obey their husbands (Brickell, 2011), a second dimension of victim blaming relates to the purported submissive tendencies of Cambodian women. In the *Chbap Srei*, a woman is instructed to move quietly around the house, be polite, avoid vulgarity, and be careful to preserve the dignity and feelings of her husband despite any indiscretion on his part (Verses 12, 115, 117, and 148, cited in Pou, 1988). Yet obedience to prescriptive codes of conduct is not necessarily rewarded with a harmonious or violence-free household. Equally, violations of gender role prescriptions do not always evoke anti-victim attitudes from institutional stakeholders. In the majority of interviews, women are actually considered too tolerant and “blindly loyal” to their spouses.

Rotha is a female commune-level deputy police head in her early 50s. She receives reports of DV from village police and is receptive to these believing strongly that women should seek immediate help. Although she holds progressive attitudes to rights claiming, her own complicity in victim blaming is linked to women’s supposed submissive behavior to their husbands. A degree of dissonance, however, was evident when analyzing Rotha’s interview. Although at the start of the interview, she emphasizes the need for women in particular to be patient, later on, this trait is critiqued:

I think it takes every single person in the family to have patience with one another, especially when one of them does something wrong. I think that the woman must be more patient because men tend to do the most wrong. Therefore, the woman must be patient and forgive the other member.

I think that it tends to be the women who prolong this type of situation at home. When she experience things like that, she keeps it to herself and allows the perpetrator to continue his abusive action. Also, when she actually reports the case to the authority and we make an arrest, she will come forward and beg for his release. These are the main reasons why these men never stop abusing their spouses because they know that the women condone their behavior. It gives them the upper hand on the matter at home. They have complete control over the affairs of the entire household . . . women here are too soft. They know what is right but refuse to stand up for it . . . .

Cognizant with the institutional trend identified by a NGO report on DV in Cambodia (Lim, 2009), Rotha initially suggests that it is women to whom patience and tolerance must fall. Yet she then moves on to highlight victims’ collusion in their own victimization either by remaining silent about the abuse suffered or by contesting arrest. Rotha again registers the significance of women’s emotionality. She criticizes women for being too “soft”—for not being more overt and assertive in their spousal demands in the domestic sphere. Her interview fails to capture the complex experience of battered women with her narrative conjuring “a new kind of deviance in the form of pathetic victims” (Dunn & Powell-Williams, 2007, p. 983). Referring back to the theme of impunity, this results in a situation where men supposedly have “the upper hand” and “complete control.” By uttering, “They know what is right but refuse to stand up for it,” Rotha places women in an agentic position in which they actively decline the upholding of justice (nominally) provided by enforcement officials. As Dunn (2010) suggests with respect to DV cases, women are often subject to criticism on two opposing fronts: both for their exertion and rejection of agency. This complexity reflects the reality in which women are not “compelled, unreflexive performers of dominant discourse(s)” (L. Nelson, 1999, p. 349) but rather more complex actors who (re)-negotiate gendered expectations in their response to DV. The “double bind” is not only implicated in Rotha’s interview but also played out in others, including that of a female provincial official who initially foregrounds the transgression of gender norms.

The wife was a bit too nagging and would yell at her husband all the time. She even dared him to hit her and stuff. One day, he got too upset about it and just picked up a long piece of sugarcane and whacked her with it. She howled for help and got her husband arrested. But I know that he was a nice man. His wife then came over the next day and told me she wanted to have him released because he needed to go to work to feed the family. So, as you can see, it is not always a one-side problem.

As Maly intimates, a DV victim is culpable to concurrent criticism. She is both too “nagging” and assertive—provoking abuse by her “nice” husband—and at the same time, making demands for his release on arrest. As Aisyah and Parker (2014) note in Indonesia, “domestic violence indexes not only the cultural construction of power within marriage, but also, ironically, wifely agency” (pp. 213-214). In other words, the problem of DV is not the sole preserve of the perpetrator but rather the agency of the victim also enters the equation. Yet toward the end of the interview when turning to the topic of policy-making, Maly showed a comprehensive understanding (much like Kamol earlier) of the underlying constraints in dealing successfully with DV:

It is always the women. There are quite a few problems associated with women when it comes to dealing with DV. First of all, she is entirely dependent on her spouse for all her financial support. Without him, she is completely on her own . . . the realization of one’s husband being imprisoned is too much for them to bear. In so doing, she gives him even more reason to abuse her. Another important issue is the notion of shame. A divorced woman in a Cambodian culture is often shunned and kept away from the rest of the community. But personally, I think that most important issue is the lack of facilities that would assist the abused women in their times of need. It is always a good idea to separate both parties up when DV occurs. It is hard for a woman to come to us for help only to be told to go home and wait.

Although at the start of the excerpt, a language of blame is still directed toward victims, Maly outlines a range of obstacles to women’s expression of agency in circumstances of abuse. Of particular note is the reference made to the paucity of safe houses, which means that a woman’s ability to “become more vocal about her mistreatment” is curtailed, and the fears of retaliation heightened as she is turned away. This reference made in Maly’s interview highlights the fallacy of the idea that individual actions taken by women are sufficient in themselves to put an end to a violent relationship. Yet, although structural support is clearly fundamental in preventing the abandonment of help-seeking actions, this section has shown how in multiple ways, victims are simultaneously blamed for withholding and exerting agency in their domestic and extra-domestic dealings, respectively.

## Conclusion

This article has explored the in-depth narratives of institutional stakeholders on the theme of victims’ reneged use of DV Law. It has shown how the viewpoints and related practices of both male and female interviewees have the potential to aggravate a climate of victim blaming. Two explanatory discourses were identified for the continued reliance on reconciliation and counterpart lack of cases entering the judicial system: first, the emotionality of women considered irreconcilable with the rationality of law, and second, the acquiescent traits of married women. A large swathe of institutional stakeholders in the study contend that female victims are not only “clouding the judgment of law” but also stifling their ability to bring perpetrators to justice. Yet, if “women’s empowerment is about the process by which those who have been denied the ability to make strategic life choices acquire such an ability” (Kabeer, 1999, p. 435), it is DV Law and its enforcers that are failing women rather than the other way around. With respect to legal reform, for example, Engle Merry (2006) aptly comments that

it is not surprising that women would adopt a tentative stance towards this transformation—try it on, dropping it, trying it again . . . Women’s ability and willingness to move into this subjectivity depends, of course, on how the law treats them. (pp. 189-190)

Acknowledging, yet ultimately downplaying, the structural factors that materially curtail women’s access to justice, the rhetorics analyzed in the article highlight the conflict between victimization and agency in representations of women (Dunn, 2005). In these, “not all constructions bear equal weight, and those that prevail have perhaps unintended consequences” (Dunn & Powell-Williams, 2007, p. 980). In Cambodia, women are, on balance, painted as agents in their own continued victimization. In turn, they are also construed as complicit in a pervasive culture of impunity. Investing women with agency not only operates as a means to resolve institutional stakeholders’ professional disempowerment through the inadequacies of state resource provision, but also works to mask their own collusion. The article thereby signals the importance of research on, and better professional support of, intermediaries who (discursively) administrate the relationship between victims/citizens and the governments who ratify, yet are unsuccessful in creating the conditions for women’s legal rights to be meaningfully actioned. Support means more than just training on gender sensitization or the technical details of Cambodia’s DV Law; rather, it also requires local stakeholders to have their legal obligations properly budgeted and resourced so that they can advocate on a victim’s behalf in an enabling institutional environment.

## Notes

1. To protect the anonymity of respondents, where the research was conducted is not named beyond the provincial reference given in the methodology. Participants’ names and exact job titles for provincial authorities who could be identified have also been changed.

2. Since this point, a range of academic research has been published on domestic violence (DV) in Cambodia relating to the “triple burden” of disability (Astbury & Walji, 2014), nongovernmental organization (NGO) programming (Lilja, 2011), refuge in Buddhism (Kent, 2011), experiences of marriage (Surtees, 2003; Yount & Carrera, 2006), and alcohol as an external explanation for DV (Brickell, 2008).

3. Local administration is represented at two levels: the province and the district. Each district consists of a group of communes (which are themselves composed of different village clusters).
